# Supplementary material for: Association between oral microbiome and seven types of cancers in East Asian population: a two-sample Mendelian randomization analysis
Source: Front Mol Biosci. 2023 Nov 21;10:1327893. doi: 10.3389/fmolb.2023.1327893 (PMC10702768; doi:10.3389/fmolb.2023.1327893)
Supplement: Supplementary file 1 [file DataSheet1.ZIP › Supplementary Materials/MR plots for tongue/tongue═╝/Prostate cancer/pheno.699_to_prostate cancer_leave_one_out.pdf]

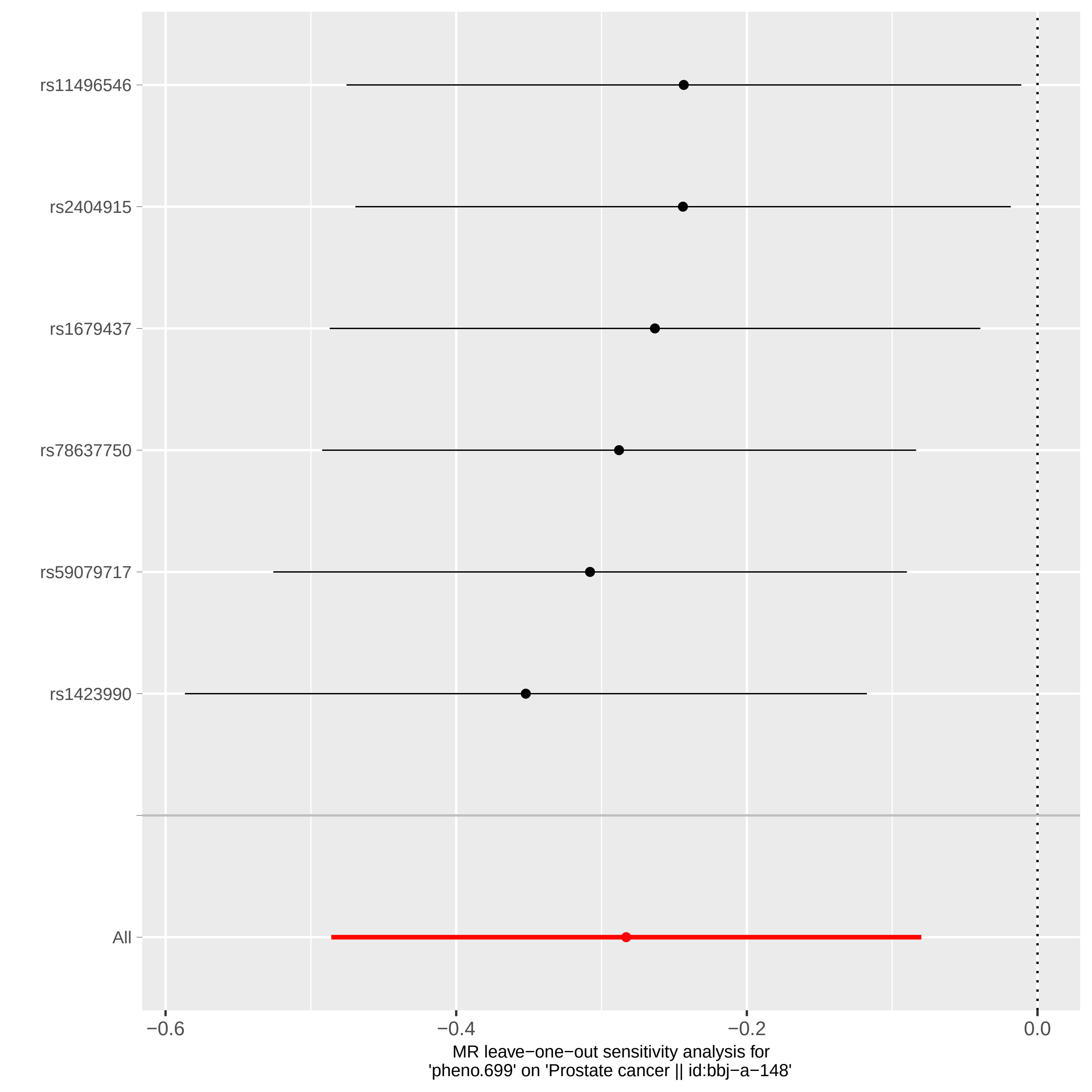
rs11496546
rs2404915
rs1679437
rs78637750
rs59079717
rs1423990
All
−0.6
−0.4
−0.2
0.0
MR leave−one−out sensitivity analysis for
'pheno.699' on 'Prostate cancer || id:bbj−a−148'
